# Properties of Phosphorus-Slag-Based Cementitious Pastes for Stabilizing Lead

**DOI:** 10.3390/ma12233831

**Published:** 2019-11-21

**Authors:** Xuquan Huang, Liang Liu, Xiaorong Zhao, Cilai Tang, Xiaoshu Wang

**Affiliations:** 1 College of Hydraulic and Environmental Engineering, China Three Gorges University, Yichang 443002, China; huangxuquan@126.com (X.H.); taoyi.2007.cool@163.com (L.L.); rongrong315@126.com (X.Z.); bolong@ctgu.edu.cn (C.T.); 2 Hubei Engineering Technology Research Center for Farmland Environmental Monitoring, China Three Gorges University, Yichang 443002, China; 3 Engineering Research Center of Eco-environment in Three Gorges Reservoir Region, Ministry of Education, China Three Gorges University, Yichang 443002, China

**Keywords:** phosphorus slag, lead, solidification, stabilization

## Abstract

The properties and curing mechanism of leaded samples solidified with phosphorous-slag-based cementitious pastes are studied. The compressive strength, pH of percolate, and lead-ion concentrations of leaded samples stabilized with the phosphorous-slag-based cementitious pastes and cement were analyzed. Results confirmed that the phosphorous-slag-based cementitious paste performed much better than cement in terms of solidifying lead. The cured form of lead with phosphorous-slag-based cementitious pastes had higher compressive strength, lower lead leaching, and smaller change in pH. Higher lead content corresponded with more obvious advantagees of phosphorus-slag-based cementitious pastes and lower risk of environmental pollution. By means of X-ray Diffraction (XRD), Fourier Transform Infrared Spectroscopy (FTIR), and Energy Dispersive Spectrometer-Scanning Electron Microscope (EDS-SEM) analyses, we found that the hydration of phosphorus-slag-based cementitious pastes produced hydrated calcium silicate gels, ettringite and other minerals with large specific surface areas, as well as some leaded products that can combine with lead ions to form chemically stable leaded products. This finding well explained the high performance of phosphorus-slag-based cementitious pastes in terms of lead solidification and stabilization.

## 1. Introduction

Lead, which is widespread in soils contaminated with heavy metals, has strong accumulation capacity and strong biological toxicity [1]. This metal originates mostly from mining [2], metal smelting and processing, sewage discharge, sewage irrigation, and the use of leaded gasoline; thus, soil pollution by lead has become a primary concern. Cement-based solidification/stabilization process is extensively used to control heavy-metal pollution [3], but this process has the drawbacks of relatively high volume-change ratio, high cement cost, unstable solidified products [4] and environmental-related problems during cement production [5,6].

Phosphorous slag is a by-product of granular industrial production in the yellow phosphorus industry. Generally, 8–10 tons phosphorous slag is produced for every 1 ton of yellow phosphorus product [7]. For instance, the amount of phosphorous slag exceeds 8.0 million tons annually, so the accumulation and pollution of phosphorus slag has become a serious problem in China [8]. The main components of phosphorus slag are calcium phosphate, pseudo-wollastonite, quartz, apatite, cuspidine, tricalcium silicate, and dicalcium silicate [9,10]. Building-material scientists have explored many application approaches of phosphorus slag as cementitious pastes for producing high-performance concrete [11], but very few have focused on using phosphorus slag in the solidification/stabilization of lead-containing soil. Li et al. [12] found that phosphorous slag could effectively reduce the leaching concentration of lead in the cured form compared with cement-solidified leaded soil. The researchers have solidified lead-contaminated soil with the binder consisting of 70% ordinary Portland cement and 30% phosphorus slag at a binderto-dry soil ratio (wt./wt.) of 0.5. After curing the solidified samples at 20 ± 1 °C and 95% Rh, the concentration of Pb leaching by acetic acid decreases to 1.98 mg/L after 28 days of aging. However, the curing agent has 70% cement, and thus remains cement in essence, so the toxicity of leaching solution and the changes in microstructure require further investigation. Phosphorus slag contains small amounts of calcium phosphate and apatite that can solidify/stabilize leaded soils [13,14]. Accordingly, the curing effect and mechanism of phosphorus-slag-based cementious material as an alternative cement-curing agent for lead-contaminated soil needs further study in order to expand phosphorus-slag application and contribute to our knowledge on regarding disposal of phosphorous slag.

In the present study, phosphorus slag was mixed with slag, clinker, and a small amount of chemical activator as curing agent for the heavy metal lead. The curing effect of phosphorus slag was compared with that of cement. The unconfined compressive strength, lead concentration and pH change of the solidified body were studied. The hydration products of lead samples stabilized with phosphorous-slag-based cementitious pastes were analyzed with X-ray Diffraction (XRD), Fourier Transform Infrared Spectroscopy (FTIR), and Energy Dispersive Spectrometer-Scanning Electron Microscope (EDS-SEM). The results may confirm the phosphorous-slag-based cementitious pastes’ good solidification ability for lead and reveal the underlying mechanism through microscopic analysis of the hydration products. The findings may also serve as a basis for further applying phosphorus slag as cementitious pastes with high efficiency.

## 2. Materials and Methods

### 2.1. Solidified Materials

The curing agents used were phosphorus-slag-based cementitious pastes (PS) and P•C 32.5 cement (PC). PS was composed of 50% phosphorus slag (PP), 17% blast furnace slag (BS), 25% clinker (CC), 2% sodium hydroxide, 3% sodium sulfate, and 3% sodium silicate. PC was obtained from Gezhouba Group Cement Corp. Ltd. (Gezhouba, China). By using a specific-surface-area analyzer (Mastersizer 2000, Malvern Panalytical, Malvern, England), the Blaine’s surface areas of PC, PP, BS, and CC were determined to be 340.1, 502.4, 321.3, and 334.6 m^2^/kg, respectively. The chemical compositions of the original materials are shown in Table 1.

### 2.2. Experimental Methods

#### 2.2.1. Stabilization Experiment

Each component of PS as the previous formula of PS curing body was weighed. A volume of lead nitrate and distilled water was added into the mixture of PS components according to a given mass ratio 0.4 of water/cement. The resulting lead-ion concentrations (mass ratios) in the mixture was 0%, 0.31%, 0.63% and 1.26%, respectively. The prepared mixture was placed in a mortar mixer and stirred for 3 min. All pastes were casted into 40 mm cubic molds and vibrated for 30 s in a flat-panel vibration machine before curing at 20 ± 1 °C and 95% Rh for 24 h. After demoulding, the cubic specimens were cured under water at 20 ± 1 °C to the provisions of aging. These samples’ compressive strengths were determined at predetermined ages according to the Chinese Standard “Method of testing cements–Determination of strength” (GB/T 17671-1999) [15]. 

#### 2.2.2. Leaching Toxicity Experiment 

The solidified body was dried to a constant weight at 60 °C and crushed below 3 mm. The samples and deionized water were weighed and mixed according to a mass ratio of 1:10. The percolate was prepared according to the Solid waste-extraction procedure for leaching toxicity-Acetic acid buffer solution method (HJ/T 300-2007). The pH of percolate and the concentration of total lead were measured. 

#### 2.2.3. Microstructure Analysis

The solidified body was taken out after being immersed in absolute ethanol for 48 h, and dried at 60 °C to a constant weight for XRD, FT-IR, and SEM analyses. A scanning electron microscope (JSM-5610LV, JOEL, Tokyo, Japan) united with the Energy Dispersive Spectrometer (Phoenix, EDAX, Mahwah, NJ, America) were used to scan the micromorphology and analyze quantitatively the contents of elements. The XRD patterns of crushed samples were obtained on a Rigaku Ultima IV diffractometer (Ultima IV, Shimadzu, Kyoto, Japan) with a Cu target. FT-IR spectra were recorded on a Nicolet iS 50 Fourier transform infrared spectrometer (Nexus, Thermo Nicolet, Waltham, MA, America).

## 3. Results and Discussion

### 3.1. Solidification Properties

**Figure 1:**
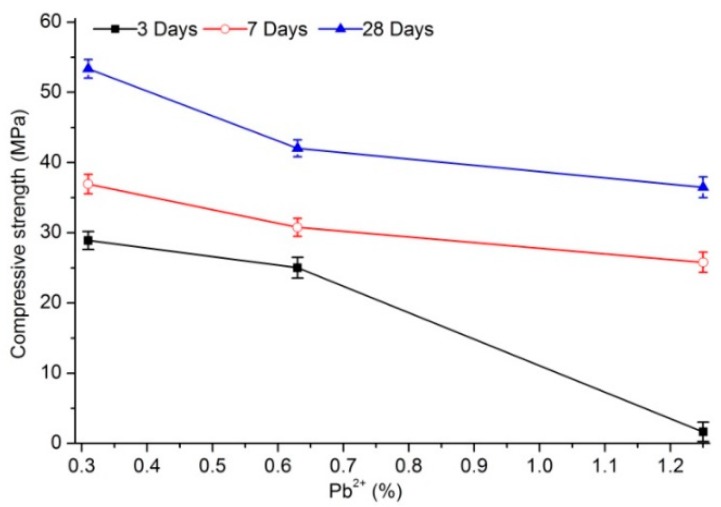
Compressive strength of samples cured with P•C 32.5 cement (PC).

**Figure 2:**
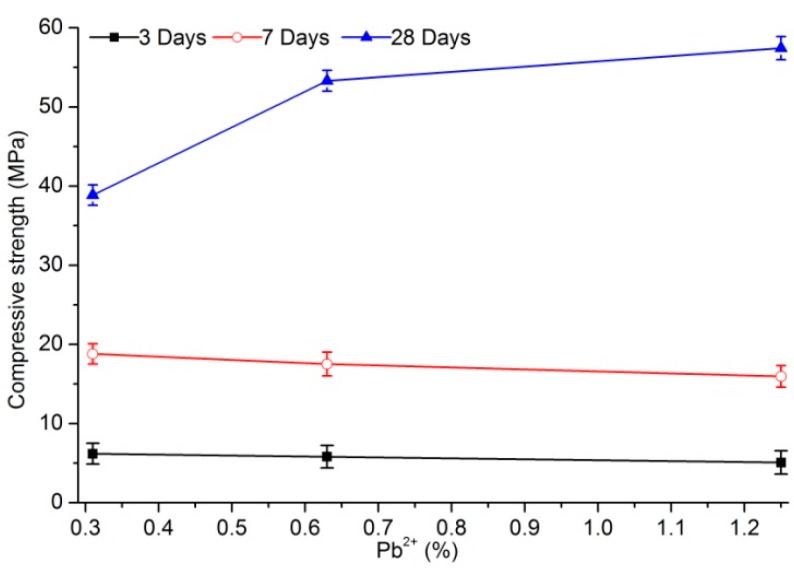
Compressive strength of samples cured with phosphorus-slag-based cementitious pastes (PS).

The compressive strength of samples doped with the same lead content increased with aging regardless of PC or PS as curing agent (Figure 1 and Figure 2). With increased lead content from 0.31% to 1.26%, the compressive strength of samples cured with PC decreased from 28.9, 36.9, and 53.4 MPa to 1.65, 25.8, and 36.5 after 3, 7, and 28 days of aging, respectively. The variations in compressive strength of the samples with the different lead contents ware −94.3%, −30.1%, and −31.6%, respectively, among which the compressive-strength reduction after 3 days of aging was the most prominent. However, with a relatively short aging time, lead content did not contribute to the compressive strength of leaded samples cured with PS. In particular, the compressive strength of leaded samples stabilized with PS remained constant after 3 or 7 days of aging with increased lead content. When the aging of leaded samples cured with PS lasted until day 28 of aging, the change trend of compressive strength appeared to be significant. The 28-day compressive strength of samples with 0.31% lead content was 38.9 MPa, whereas 0.63% and 1.26% lead content resulted in 1.37 and 1.48 times the compressive strength, respectively. Thus, the compressive strength of the cured samples after long-time aging can be improved by increasing the lead content. 

**Figure 3:**
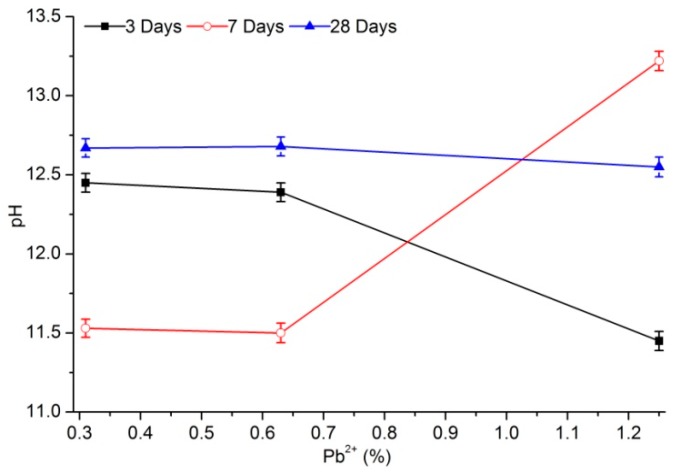
pH of percolate from samples stabilized with PC.

**Figure 4:**
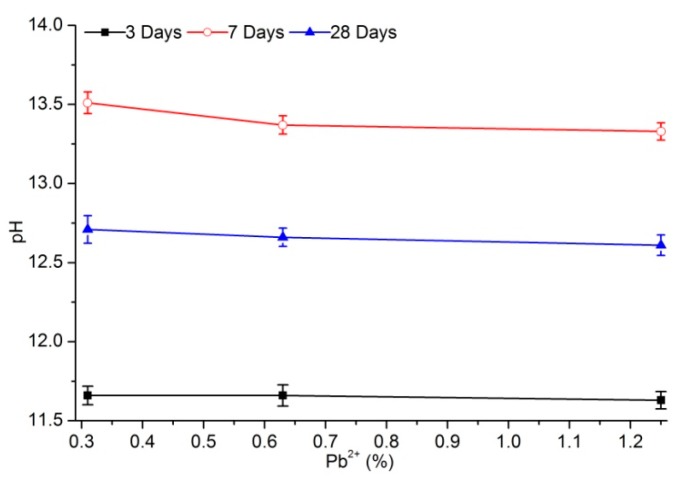
pH of percolate from samples stabilized with PS.

Figure 3 and Figure 4 show the change in pH with lead content for different aging times. With increased lead concentration in the sample, the pH of PS solidified sample percolate remained nearly unchanged, which may benefit situations wherein the actual lead-pollution concentration greatly fluctuated. However, the pH of percolate from samples stabilized with PC greatly fluctuated, which was not conducive to lead stability and adsorption. The pH of percolate from samples cured by PS with the same lead content were higher than those cured by PC for different curing ages except for the samples aged of 3 days. After 3 days of aging, the pH of percolate from samples cured with PS averaged at 11.7 and hardly fluctuated, whereas that cured with PC averaged at 12.1 and decreased with increased of lead-ion content. After 7 days of aging, the pH of percolate from samples stabilized with PS was 13.4 on average, whereas that of PC was 12.1.

After 28 days of aging, the pH of percolate from samples stabilized with PS exceeded 12.6, whereas that stabilized with PC was between 12.55 and 12.68. The possible reason for this was that the amount of lead adsorbed by the hydration products of PS was more than that of PC. Many hydroxide products were present in samples stabilized with PC, so the alkalinity of the cured body may have decreased [16]. The pH values of the two kinds of cured samples after 28 days of aging were relatively close, and remained stable with the change in content, indicating that most absorbable lead and precipitated lead had achieved stability.

"N/A" means that the concentration of lead in the percolate is lower than the limit of detection. For the original sample with high concentration of lead, the lead concentration in the percolate from the solidified body cured with PC or PS decreased significantly. According to Table 2, when the content of Pb^2+^ was 0.31%, the lead concentration in the percolate of solidified body cured with PC at 3 days of aging was 2.61 times of that cured with PS. The lead concentration in the percolate from cured body that stabilized after 7 days of aging was significantly larger than that stabilized after 3 days of aging, whereas the lead concentration of cured body stabilized with PC was 7.65 times that cured with PS. Conversely, when the initial lead concentration was 0.63%, the lead concentration in the percolate from the cured body with PC after 3 days of aging was only 24.0% of that cured with PS. The lead concentration in the percolate of solidified body cured with PC after 7 days of aging increased by 10.2 times compared with that after 3 days of aging. However, the lead content in the percolate of solidified body cured with PS after 7 days of aging decreased to 43.9% compared with that after 3 days of aging. The lead concentration stabilized with PC was 6.13 times that stabilized with PS.

After 3 days of aging, the lead concentration in the percolate of PC solidified body was higher than that of PS only except when the initial lead content was 0.31%. These findings may be due to the significantly higher calcium hydroxide produced by PC hydration than PS at early-stage curing. The lead precipitation reaction developed effectively in the presence of calcium hydroxide, inducing low lead concentration in the percolate of cured body with PC. This phenomenon was in accordance with our finding that the pH of percolate from PC-cured body was slightly higher than that from PS-cured body. It was also related to the high alkalinity of PC, the setting-retarding effect of PS, and the few hydration products at the early stage. Conversely, after 7 and 28 days of aging, the lead content of the percolate from the cured body solidified with PS was lower than that with PC at a given initial lead concentration, which may have resulted from the numerous generated adsorptive products with strong adsorption capacity from the series of reactions. These products further adsorbed a large amount of residual lead.

Compared with PC, PS showed an overall higher lead-solidification performance, which became more noticeable with higher lead contents.

### 3.2. XRD Analysis

**Figure 5:**
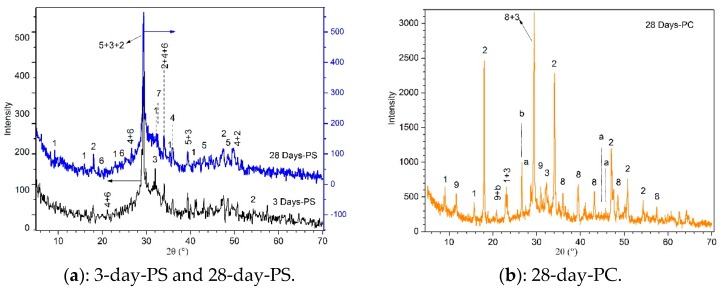
X-ray Diffraction (XRD) patterns of leaded solidified samples at different hydration ages. 1-Ettringite, 2-Portlandite, 3-Calcium Silicate Hydrate, 4-Lead carbonate hydroxide hydrate, 5-Calcite, 6-Hydrocerussite, 7-Larnite, 8-Calcite, 9-Gypsum, a-Lead Hydroxide, b-Quartz. (**a**): 3-day-PS and 28-day-PS, (**b**): 28-day-PC.

Figure 5 shows the XRD patterns of 1.26% lead samples stabilized respectively with PS and PC at different hydration ages. As seen from Figure 5a, substances such as Portlandite (Ca(OH)_2_), hydrated calcium silicate gels (Ca_6_Si_3_O_12_·H_2_O), calcite (CaCO_3_) were detected at day 3 hydration age [17], indicating that they easily formed in the early stages. With prolonged hydration time (28 days), the characteristic peaks of ettringite (Ca_6_Al_2_(SO_4_)_3_(OH)_12_·26H_2_O) were significantly enhanced, indicating ettringite was still continuously generated. The characteristic peaks of calcium silicate hydrate gels were obviously weakened and larnite (Ca_2_SiO_4_) was simultaneously detected, which may be ascribed to crystal phase transition of calcium silicate hydrate. Along with the decrease in characteristic peaks of calcite, the peaks of hydrocerussite (Pb(CO_3_)_2_(OH)_2_) were enhanced noticeably. Meanwhile, the characteristics peak of lead carbonate hydroxide hydrate (3PbCO_3_·2Pb(OH)_2_ ·H_2_O) was slightly developed [16]. By contrast, it can be seen from Figure 5b that the cured products from pure cement solidified lead after 28 days mainly exist in the form of lead hydroxide [18], which is chemically unstable and easily dissolved in strong alkaline solution or carbonized. Certainly, the hydration products cured with PC also include substances such as calcium silicate hydrate, ettringite and so on, which can absorb lead. These might be the main reason that cement can solidify lead but the solidification effect is lower than phosphorus slag solidified lead compared with Figure 5a. These phenomena may involve a series of complex mineral-recombination reactions, which can drive calcite transform into compounds such like lead carbonate hydroxide hydrate, hydrocerussite, and others containing elemental lead.

### 3.3. EDS-SEM Analysis

**Figure 6:**
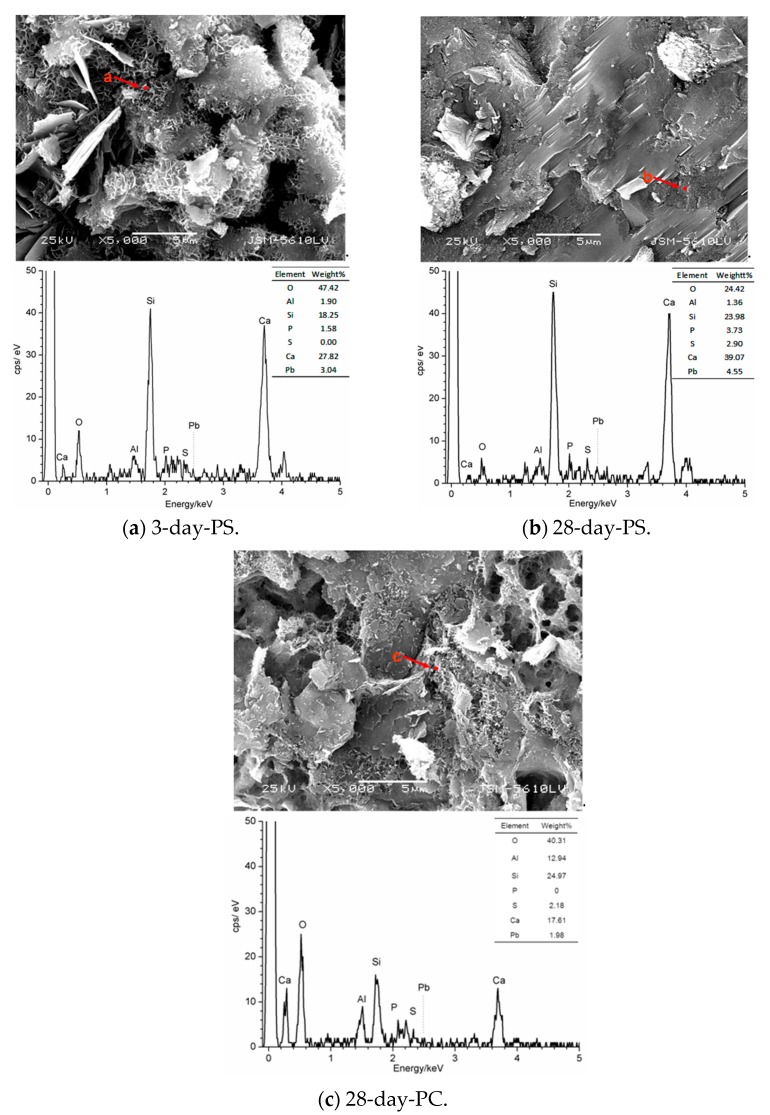
EDS-SEM micrographs of leaded solidified samples at different hydration ages. (**a**) 3-day-PS, (**b**) 28-day-PS, (**c**) 28-day-PC.

The SEM image (Figure 6) clearly revealed the pores and spaces between the hydration products in the microstructure, indicating that the filiform hydrated calcium silicate and lamellar hydrated calcium hydroxide constituted a spatial network. In order to verify the solidification results of leaded solidified samples, EDS was employed to analyze the element-content distribution in the solidified materials. It can be seen from Figure 6c that the lead content of the hydrated product adsorbed by the solidified body cured with PC after 28 days of aging is only 1.98%, significantly lower than the solidified body cured with PS (3.04% after 3 days and 4.55% after 28 days). Points a, b and c in the samples with different hydration ages were respectively taken and marked in the images.

**Figure 7:**
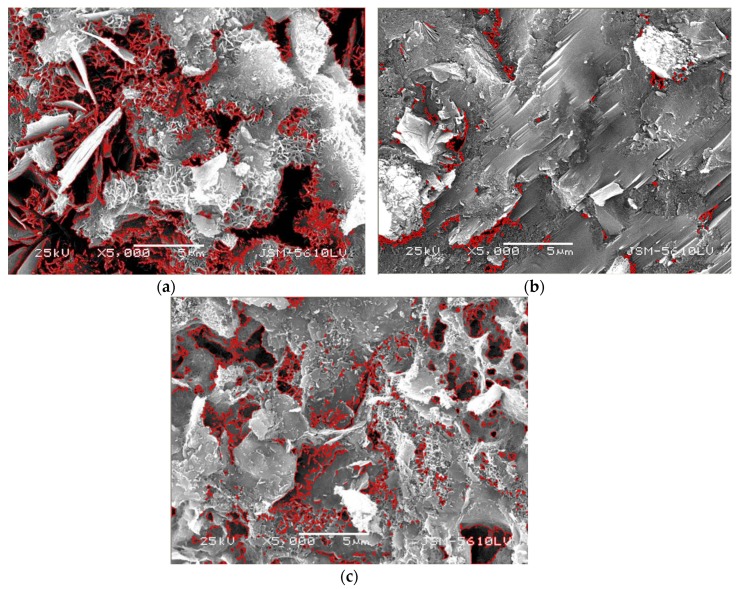
Calculation diagram of the porosity. (**a**): 3-day-PS, (**b**): 28-day-PS, (**c**): 28-day-PC.

The 3-day-hydrated calcium silicate gel contained a certain amount of elemental lead, indicating that lead can replace elemental calcium in hydrated calcium silicate gel [19] or enter its microstructure in ionic form [20]. The lead content of the crystallized hydrated calcium silicate may be higher after 28 days. In terms of sulfur elements, lead ions may have also been displaced into the lattices of ettringite [21]. The above microstructure photos for different hydration ages (Figure 7a,b) were analyzed with Image Pro-Plus software, and the porosity of the 3 day-hydrated sample was 31.99%, whereas that of the 28 day-hydrated sample was 2.06% [22]. However, compared with the solidified body cured with PS, the porosity of the 28-days solidified body cured with PC is 13.38% (Figure 7c). We further concluded that the compactness of the microstructure of samples hydrated for 28 days increased, thereby increasing the difficulty of lead leaching. Consequently, the leaching concentration of lead in the 28 day-hydrated sample was lower than that of the 3 day-hydrated sample.

### 3.4. FTIR Analysis

**Figure 8:**
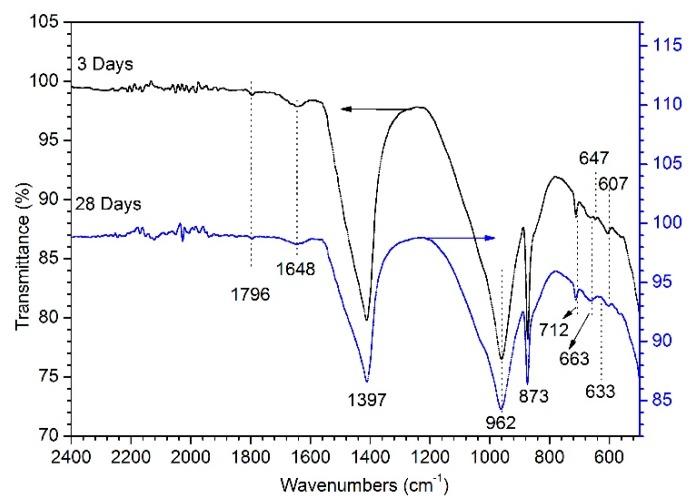
FT-IR spectra of leaded solidified samples at different hydration ages.

In order to further discuss the stability of leaded solidified samples cured with PS at different hydration ages, the microsurrounding of the sample structure was studied by FT-IR spectroscopy, which was exhibited in Figure 8. In the solidified samples, an additional spectroscopic feature was related to the presence of γ2-H_2_O at 1648 cm^−1^ assigned to deformation water mode in ettringite [23]. The peaks at 607 and 633 cm^−1^ were also due to the SO_4_^2−^ bending vibration in ettringite [24]. The band at 876 cm^−1^ was due to the symmetric and asymmetric vibrations of Al–OH band in the Al(OH)_6_ octahedral structure of ettringite [25]. The absorption bands at 962 and 669 cm^−1^ were due to the Si–O–Si vibrations of Q^2^ tetrahedral, indicating the formation of calcium silicate hydrate gel [26,27]. At different hydration ages, absorption bands at 712 and 1397 cm^−1^ were observed and attributed to the formation of the –CO_3_ group, which indicated the presence of lead carbonate and thus the formation of lead carbonate hydroxide hydrate or hydrocerussite [28,29]. The absorption band at 1796 cm^−1^ remained basically unchanged throughout the hydration phase corresponding to stretching vibrations of the –CO_3_ group, which proved the presence of calcite [30].

## 4. Conclusions

Lead content significantly affected the compressive strength of cured body stabilized with PC. A higher lead content corresponded with lower strength. However, the compressive strength of cured body stabilized with PS showed a different pattern. The change in strength of the solidified body with increased lead content of the sample aged for 3 days was not apparent. By contrast, the change in strength obviously increased with increased lead content of the sample aged for 28 days. 

Regardless of lead solidification with PC or PS, the pH of percolate from solidified samples remained stable at day 28 of aging with increased lead content, which guaranteed the stability of adsorption or precipitation of lead. However, the pH of percolate from samples solidified with PC varied greatly with different initial lead contents, whereas lead content exerted little impact on those solidified with PS, indicating its superior solidification stability. 

The long-term stability of alkalinity can ensure the stability of hydration products and structures of solidified bodies, and the risk of lead-leaching contamination in solidified body with PS was significantly lower than that with PC. 

PS was hydrated to produce hydrated calcium silicate gels, ettringite, and other minerals with large specific surface area. At the same time, some ingredients can combine with lead ions to form leaded products with stable chemical properties, which was the main reason for the high performance of PS in terms of curing and stabilizing lead. Moreover, the increased compactness of microstructure with increased age also contributed to the greater solidification/stabilization of lead in cured body solidified with PS.

Based on the present findings, further studies on the long-term performance of solidified body under acid rain or CO_2_ erosion, and the solidification/stabilization of actual lead-contaminated soil and waste residue can benefit the high-quality utilization of phosphorus slag and serve as a basis for new soil-remediation technologies.

## Figures and Tables

**Table 1:** Chemical compositions of the main raw materials (mass ratio, %).

Materials	CaO	SiO_2_	Al_2_O_3_	MgO	SO_3_	Fe_2_O_3_	P_2_O_5_	F
PP	45.3	42.1	3.31	1.77	--	1.31	3.67	2.49
BS	38.7	29.9	15.9	8.0	3.7	1.2	--	--
CC	65.5	20.0	4.4	1.6	1.9	3.3	--	0.3
PC	45.87	27.37	10.46	6.68	2.64	2.29	--	--

**Table 2:** Lead concentration of samples stabilized with different materials at different ages.

Lead (wt.%)	Lead Concentration of Samples Cured With PC (mg/L)			Lead Concentration of Samples Cured With PS (mg/L)		
	3 days	7 days	28 days	3 days	7 days	28 days
0.31	0.086	0.352	N/A	0.033	0.046	N/A
0.63	0.258	2.891	1.810	1.076	0.472	0.311
1.26	0.648	1.537	1.551	1.761	0.958	0.622
